# Combined Application Effects of Arbuscular Mycorrhizal Fungi and Biochar on the Rhizosphere Fungal Community of *Allium fistulosum* L.

**DOI:** 10.4014/jmb.2303.03026

**Published:** 2023-06-26

**Authors:** Chunxiang Ji, Yingyue Li, Qingchen Xiao, Zishan Li, Boyan Wang, Xiaowan Geng, Keqing Lin, Qing Zhang, Yuan Jin, Yuqian Zhai, Xiaoyu Li, Jin Chen

**Affiliations:** 1 Schools of Life Sciences, Anhui Agricultural University, Hefei 230036, P.R. China; 2 National Engineering Laboratory of Crop Stress Resistance Breeding, Anhui Agricultural University, Hefei 230036, P.R. China; 3 Anhui Province Key Laboratory of Crop Biology, Anhui Agricultural University, Hefei 230036, P.R. China

**Keywords:** Arbuscular mycorrhizal fungi, biochar, phylogenetic relationships and distribution, Illumina MiSeq high-throughput sequencing, molecular ecological networks analysis

## Abstract

Arbuscular mycorrhizal fungi (AMF) are widespread soil endophytic fungi, forming mutualistic relationships with the vast majority of land plants. Biochar (BC) has been reported to improve soil fertility and promote plant growth. However, limited studies are available concerning the combined effects of AMF and BC on soil community structure and plant growth. In this work, a pot experiment was designed to investigate the effects of AMF and BC on the rhizosphere microbial community of *Allium fistulosum* L. Using Illumina high-throughput sequencing, we showed that inoculation of AMF and BC had a significant impact on soil microbial community composition, diversity, and versatility. Increases were observed in both plant growth (the plant height by 8.6%, shoot fresh weight by 12.1%) and root morphological traits (average diameter by 20.5%). The phylogenetic tree also showed differences in the fungal community composition in *A. fistulosum*. In addition, Linear discriminant analysis (LDA) effect size (LEfSe) analysis revealed that 16 biomarkers were detected in the control (CK) and AMF treatment, while only 3 were detected in the AMF + BC treatment. Molecular ecological network analysis showed that the AMF + BC treatment group had a more complex network of fungal communities, as evidenced by higher average connectivity. The functional composition spectrum showed significant differences in the functional distribution of soil microbial communities among different fungal genera. The structural equation model (SEM) confirmed that AMF could improve the microbial multifunctionality by regulating the rhizosphere fungal diversity and soil properties. Our findings provide new information on the effects of AMF and biochar on plants and soil microbial communities.

## Introduction

Biochar is a carbon-rich biomaterial produced by pyrolysis or gasification of raw materials, such as wood or crop residues, animal and food waste, and sewage sludge [1]. It has a highly porous physical structure that increases the ability of soil to retain water and nutrients [2, 3]. As biochar is rich in carbon and plant nutrients, it can maintain soil fertility and improve crop yield [4]. Previous studies have demonstrated that biochar application improves soil fertility, carbon sequestration, and bioenergy production [5-7]. In summary, biochar application to soil can increase crop yield; however, its beneficial effects on plant growth through direct or indirect mechanisms may vary with plant species.

Arbuscular mycorrhizal fungi (AMF) are important soil microorganisms that have a symbiotic relationship with higher plants, colonizing their roots by forming clumps and vesicles [8]. As a major component of the rhizosphere microbial community in natural ecosystems, AMF can promote nutrient cycling. The beneficial effects of AMF and root symbiosis on plant growth are well documented in previous studies showing that AMF can improve plant roots and promote growth [9-12]. AMF changed the plant root structure, improving water and nutrient absorption [13]. It enables plants to obtain more soil space through the external mycelium of roots, as well as available soil phosphorus and other nutrients, increasing the photosynthesis rate of host plants [14]. Further, AMF can form symbiotic relationships among plants and regulate their mineral nutrition while promoting growth [15].

Previous studies have also demonstrated that biochar can promote AMF root colonization [16]. Biochar treatment of acidic soil can maintain pH, increase cation exchange, and preserve nutrients, promoting AMF growth [17]. Although Ohsowski *et al*. discovered that AMF symbiosis would not affect grass growth on biochar-improved soil [18], Liu *et al*. discovered that corn growth increased the most when the soil was treated with biochar and AMF simultaneously [19]. In contrast, Liu *et al*. reported that biochar diminished the growth-promoting effects of AMF on potatoes [20].

This study aims to: (1) determine the impact of AMF and biochar on the soil microbial community and *Allium fistulosum* L.; (2) evaluate the difference between AMF and biochar applied separately and simultaneously; and (3) determine the direct and indirect effects of AMF on soil carbon, nitrogen, phosphorus, fungal diversity, and microbial versatility.

## Materials and Methods

### Experimental Materials

The soil of this pot experiment was collected from the Agricultural Testing Ground of Anhui Agricultural University (31.83°N, 117.24°E). Soil properties were: carbon (1%), nitrogen (0.08%), phosphorus (0.05%), and potassium (2.1%). The collected soil sample was air-dried, gently crushed, sieved through a 2-mm nylon mesh, and stored in closed plastic containers for subsequent experiments. The *A. fistulosum* L. seeds were provided by the Life Sciences College, Anhui Agricultural University laboratory. Arbuscular mycorrhizal fungi (AMF) inoculants containing spores of *Funneliformis mosseae* (*F. mosseae*, BGC NM01A, 1511C0001BGCAM0023) were provided by the National Engineering Laboratory of Crop Stress Resistance Breeding of Anhui Agricultural University. Prior to the pot experiment, AM fungal isolates were propagated on maize (*Zea mays* L.) plants in a soil/sand mixture (1:1, w/w) for 120 days. Each gram of inoculum contained approximately 5 to 10 spores. Fungal inoculants included AMF spores, mycelium, sand, and allium root fragments. Rice husk for biochar was produced at the Functional Laboratory for Biomass Resource Transformation and Use, Anhui Agricultural University (Anhui, China), and the biochar was made by pyrolysis of rice husks at a temperature of 800°C for 4 h.

### Pot Experiment

The potting experiment was carried out in the greenhouse of the Nongcui Garden at Anhui Agricultural University. The experiment was arranged with three groups: the first group was untreated and set as the control (CK), the second group received 60 g of AMF (AMF), and the third group received 60 g of AMF and 3% of biochar (AMF + BC). There were three replicates for each treatment. Ten uniformly sized *A. fistulosum* L. seeds were sown in each pot (diameter 17.7 cm, depth 12 cm) containing about 1,000 g of soil. The moisture content was maintained at 65% by adding deionized water during the pot experiment. Five seedlings with good growth were selected after 14 days, and the remaining seedlings were removed.

### Sample Collection

The plant shoots and roots from each pot were collected destructively after six weeks and washed with deionized water. The soil attached to the roots was removed and collected as rhizosphere soil. Fresh soil samples were immediately transported to the laboratory and gently screened through a 2-mm mesh to homogenize the soil. Plant samples were stored in a refrigerator at -20°C, and soil samples from each pot were divided into three parts: the first part was stored at -80°C for fungal community analysis; the second part was stored at 4°C to calculate the spore density per gram, and the third part was stored at room temperature for subsequent physiochemical parameter analyses. The plants' fresh weight, root length, height, and mycorrhizal colonization were measured. To measure mycorrhizal colonization of rinsed root blocks, root fragments stored in 70% ethanol were washed with tap water and decolorized in 10% KOH, followed by additional bleaching of highly pigmented roots with alkaline H_2_O_2_. Finally, the depigmented roots were acidified with 3%–4% HCl and stained with ink and vinegar solution [21].

### Soil Biological Analysis and High-Throughput Sequencing

Rhizosphere soil samples were taken for genomic DNA analysis and stored at -80°C for quality check and quantification. Each soil sample was tested for fungal DNA by ITS1F/ITS2R rDNA PCR. The generated reads data were analyzed using Majorbio Inc.'s (China) Illumina MiSeq platform (Illumina Inc., USA) after sequencing. Alkaline phosphatase and soil β-glucosidase (S- β- GC) were determined as previously described [22, 23].

Cloud-based data analytics (https://cloud.majorbio.com/), demultiplexing, and quality filtering of high-throughput sequencing data were performed according to the QIIME 2 (quantitative insights into microbial ecology) pipeline [24]. Reads were denoised using DADA2 and clustered into amplicon sequence variants (ASVs) after pruning [25]. The classification for each ASV was assigned against the UNITE version 7.2 database using an RDP Classifier. Python Nearest Alignment Space Termination (PyNAST) was used to perform sequence similarity searches for ITS gene sequences in the UNITE version 7.2 database. The ITS gene sequences were stored in the Sequence Read Archive (SRA) of PRJNA910605 (biological samples SAMN32136201-SAMN32136209 were used for Fungal ITS genetic data).

### Statistical Analysis

The keystone microbiota refers to the 100 most abundant genera in a whole sample. A phylogenetic tree was generated in MEGA-X to study the phylogenetic relationship and distribution of the first 100 fungal genera. The phylogenetic tree transformed data using natural logarithm (log10). Linear discriminant analysis (LDA) effect size (LEfSe) was used to identify significantly different components and biomarkers in soil fungal communities. LEfSe analysis can indicate both statistical significance and biological relevance. Gephi software was used to visualize the symbiotic network [26]. Zi-Pi diagrams of the connection values of all modules in the fungal ecological network are generated to explore the potential topological role of specific nodes in the network, while nodes are classified according to their Zi-Pi coefficients, and the topological role of each node is determined.

The functional groups in the fungal community were identified by the recently developed open annotation tool (FUNGuild) for functional guild annotation. Single-factor analysis of variance (ANOVA) was used to determine significance using SPSS 26.0 software (SPSS, USA). All data are expressed as mean and standard deviation. Analysis of variance and multiple comparisons were used to determine the significance of different treatments (*p* < 0.05). The main characteristics of plant growth are shown in Table 1. The structural equation model (SEM) was created using IBM SPSS Amos 26.0 software to show the direct and indirect effects of AMF inoculation on microbial versatility. Microbial versatility is expressed by the Shannon index.

## Results

### Microbial Community Composition and Phylogenetic Relationship

**Fig. 1:**
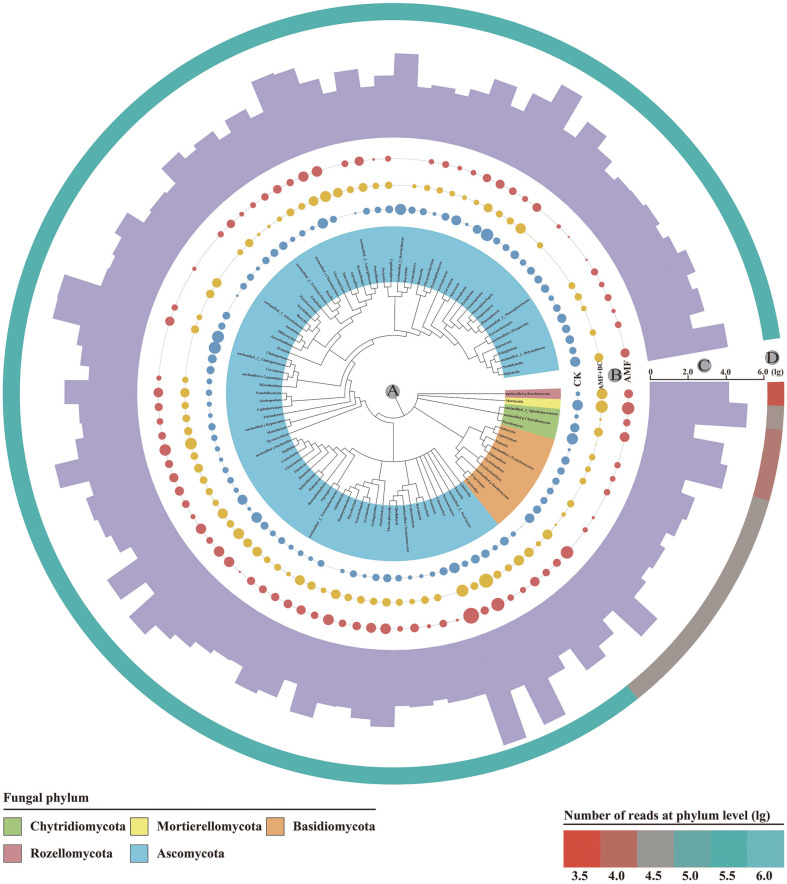
Phylogenetic relationships and distribution of the top 100 fungal genera. A: Phylogenetic tree constructed with MEGA-X and colored at the phylum level; B: Shape plot depicting the abundance of each genus; C: Simple bar chart depicting the total abundance of each genus; D: Heatmap depicting the abundance proportion of each phylum. Data were converted using the natural logarithm (log10).

MEGA-X was used to generate phylogenetic trees in this study. We selected the top 100 fungal genera with the highest abundance (Fig. 1). The keystone microbiota was divided into five phyla; Ascomycota (85 genera), Basidiomycota (10 genera), Mortierellomycota (1 genus), Rozellomycota (1 genus), and Chytridiomycota (3 genera).

### Composition and Structure of the Microbial Community

**Fig. 2:**
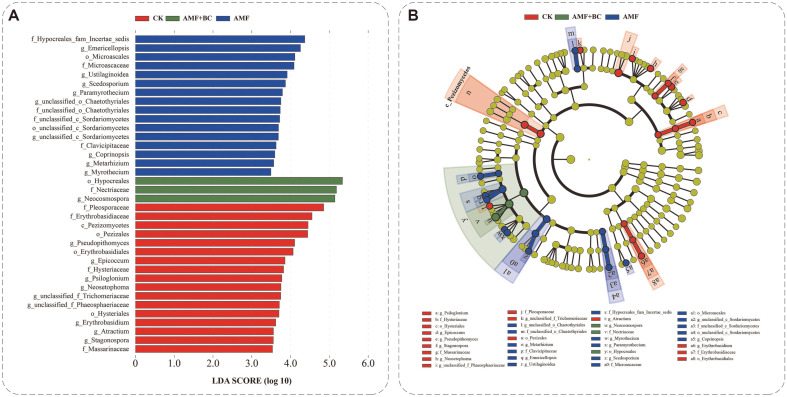
Linear Discriminant Analysis effect size (LEfSe) analysis among different treatment groups. **A**: Linear Discriminant Analysis (LDA) scores demonstrated significant group division (log10). Species with an LDA score greater than 3.0 are shown; **B**: Cladogram to indicate the phylogenetic distribution of fungi.

The effect of keystone microbiota abundance was determined using the LEfSe method. The soil keystone microbiota of *Allium fistulosum* L. crops (biomarkers) differs significantly under various experimental conditions. When the LDA threshold was 3.0, 35 fungal branches in each treatment group showed statistically significant differences (Fig. 2A). LEfSe analysis showed that 16 biomarkers were detected in the CK treatment, and 16 biomarkers were detected in the AMF treatment, while only three biomarkers were detected in the AMF + BC treatment (Fig. 2A).

The AMF + BC treatment had the highest abundance, followed by the AMF treatment and the CK. The abundance branch diagram from phylum to genus is shown in Fig. 2B; f_ Hypocreales_ fam_ *incertae_ sedis* dominated the CK. O_Hypocreales were mainly used in the AMF treatment, and f_Pleosporaceae were mainly used in the AMF + BC treatment.

### Microbial Symbiosis Network

**Fig. 3:**
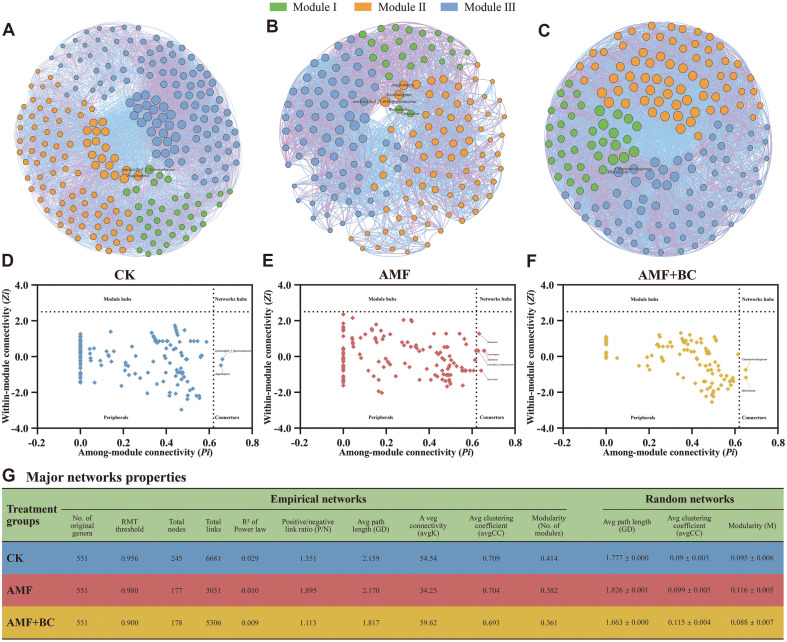
Molecular ecological network analysis representing the interaction patterns. **A, B, C**: ASV modules detected connections developed on relevance analysis of fungal genera. The size of each node is related to the extent of the connection, while the color represents different groups of samples. **D, E, F**: *Z*-*P* plots derived from topological roles. **G**: Major network properties of the three treatment groups include empirical networks and random networks.

The analysis of the keystone soil microbial symbiosis network of the three treatment groups showed different interaction modes (Fig. 3). The network was constructed using the top 100 fungal ASVs of key soil microbial communities [27]. The CK network consists of 245 nodes and 6,681 edges. The network has only 177 nodes and 3,031 edges in the AMF treatment, while there are 178 nodes and 5,306 edges in the AMF + BC treatment (Figs. 3A-3C). Figs. 3D-3F depict the *Zi-Pi* diagram showing the connection values of all modules in the fungal ecological network used to investigate the topological role of each node in the network. Different nodes play different topological roles. Node properties are classified into four types based on *Zi* 2.5 and *Pi* 0.62 thresholds: module hubs, network hubs, peripherals, and connectors. All nodes except peripherals can be called key nodes. There is no network hub in the ecological network of all fungi, and most nodes are located in peripheral devices. There were two key nodes (unclassified_f_Sporocaceae, Jugulospora) in the CK (Fig. 3D), five key nodes (Wallrothiella, Acrostalagmus, Amphotometry, unclassified_f_Arterianceae, Tetraclass) in the AMF treatment (Fig. 3E), and two key nodes (Cutaneo trichosporon, Microascus) in the AMF + BC treatment (Fig. 3F). All these nodes belong to Connectors. The RMT threshold of the symbiotic network in the CK was 0.956, and 0.980 in the AMF treatment, while the RMT threshold was 0.900 in the AMF + BC treatment. The average path length (GD), average clustering coefficient (avgCC), and modular (M) values of the empirical network are significantly higher than those of the corresponding random network (Fig. 3G). The empirical network has a higher network index than the random network, indicating that the topological properties of the empirical network are more modular and form a small world topology. The average connectivity in the AMF treatment and AMF + BC treatment was 34.25 and 59.62, respectively, compared to the CK (average K: 54.54, average CC: 0.709, P/N: 1.351, M: 0.414, GD: 2.159), while the average clustering coefficient was 0.704 and 0.693, the positive and negative link ratio (P/N) was 1.895 and 1.113, the modular (M) was 0.382 and 0.361, and the average path length (GD) was 2.17 and 1.817, respectively. The positive/negative link ratio was the highest in the AMF treatment, indicating that its positive co-current relationship was stronger than that of the other two groups. The average connectivity was higher in the AMF + BC treatment than in the AMF treatment, and included more nodes and links, indicating that the microbial community structure of its network is more complex, more modular, and has better connectivity.

### Functional Composition of Microbial Community

**Fig. 4:**
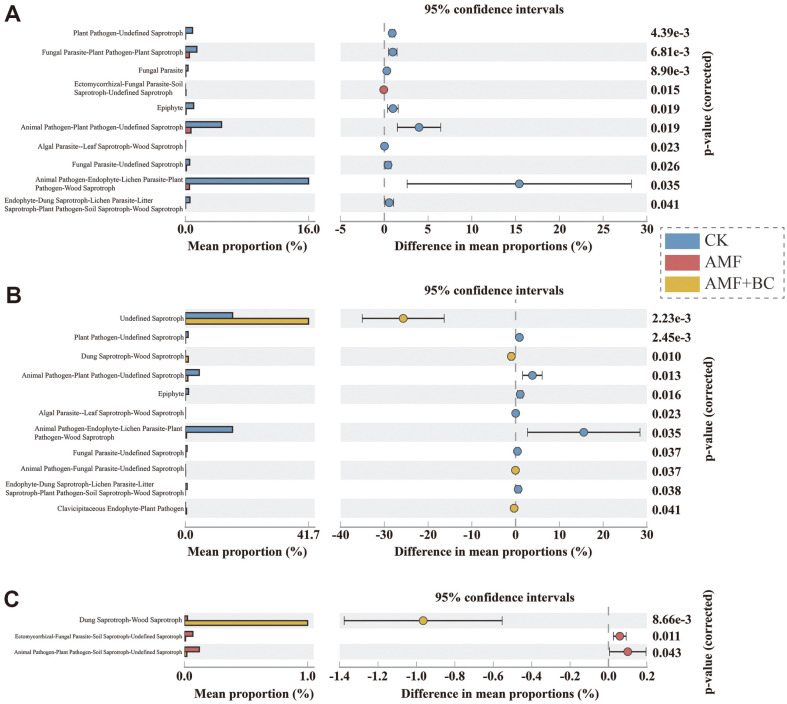
Identification of distinct treatment groups using Stamp analysis. The histogram illustrates the average proportion of various fungal genera calculated by FUNGuild (Fungi Functional Guild). The left column shows the proportion of different ASVs in three groups of samples. The middle column depicts the size of the proportion differences within the 95% confidence interval, while the rightmost column shows the *p*-value.

STAMP was used to compare the functional component abundance of different treatment groups (Fig. 4). FUNGuild (Fungal Function Association) was used to calculate the average proportion of different fungal genera. The results showed significant differences in the functional distribution of soil microbial communities among different fungi. The histogram showed that ASV abundance in the AMF + BC treatment was the highest (Fig. 4B), followed by the CK (Fig. 4A). The corresponding proportion was the lowest in the AMF treatment (Fig. 4C). There is a significant difference between undefined saprotroph and animal pathogen– endophyte–lichen parasite–plant pathogen–wood saprotroph in terms of 95% confidence interval.

### Possible Mechanisms of Environmental Parameters and Fungal Diversity Affecting Microbial Versatility

**Fig. 5:**
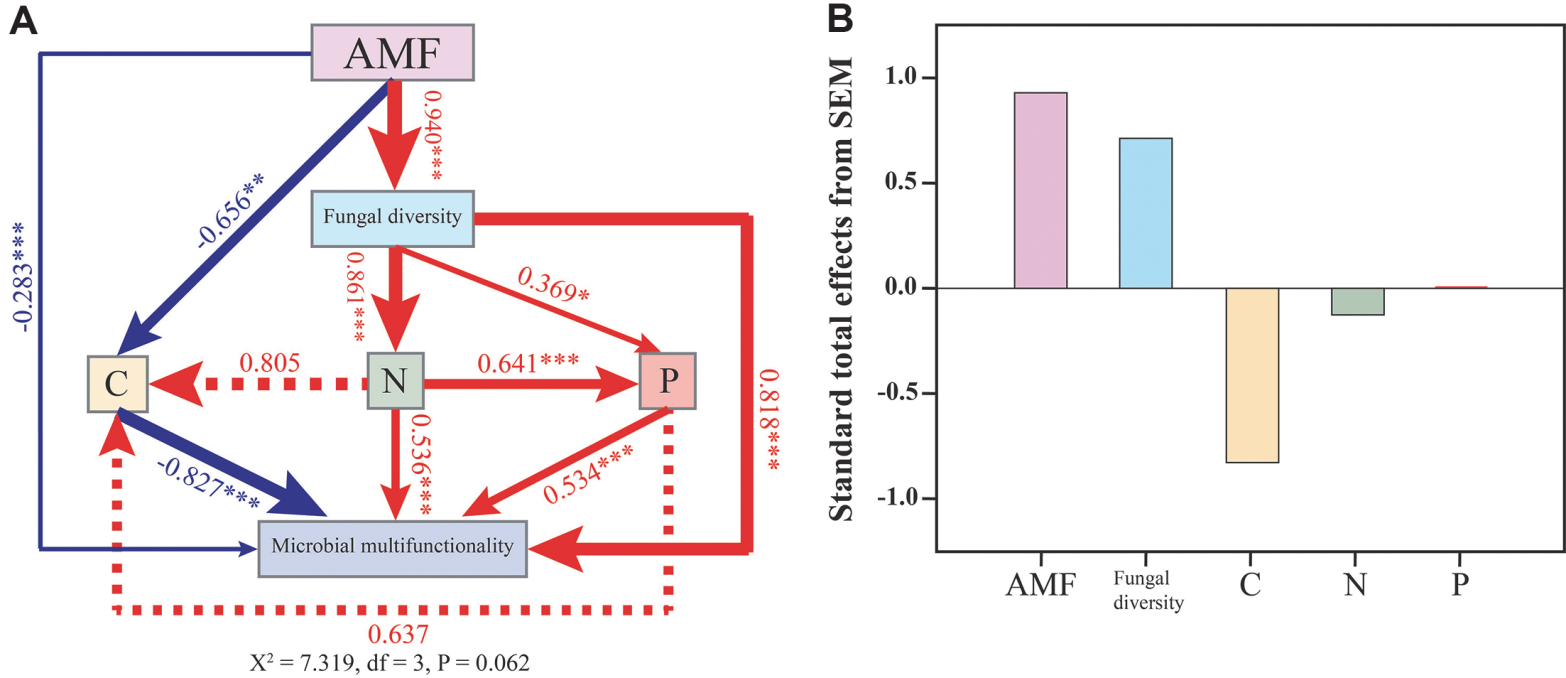
A: Structural equation models (SEMs) depicting the direct and indirect effects of AMF inoculation on microbial multifunctionality. The microbial multifunctionality is indicated by Shannon indexes. The red and blue lines represent the positive and negative relationships, respectively. The path coefficients are shown next to the arrows. Solid arrows indicate a significant relationship, while dashed arrows represent a nonsignificant relationship. Arrow widths are proportional to the path coefficients. C denotes soil β-glucosidase (S- β- GC), N denotes soil N-acetyl- β- D-glucomannase (S-NAG), and P denotes Alkaline phosphatase. Significance levels: ***, *p* < 0.001; **, *p* < 0.01; *, *p* < 0.05. **B**: Standard total effects from SEM.

A structural equation model meeting the significance criterion was developed to study the effect of AMF inoculation on the versatility of microorganisms (Fig. 5A). SEM results showed that these differences affected the microbial versatility regulation mechanism (Shannon index), which was directly affected by soil nitrogen, carbon, and phosphorus. AMF inoculation improved fungal diversity (λ = 0.940, *p* < 0.001), directly affecting soil carbon content (λ = - 0.656, *p* < 0.01) and microbial multi-function (λ= - 0.283, *p* < 0.001); soil fungal diversity and nitrogen content (λ = 0.861, *p* < 0.001), phosphorus (λ = 0.369, *p* < 0.05), and microbial versatility (λ = 0.818, *p* < 0.001). There is also a relationship between the concentrations of three enzymes representing soil carbon, nitrogen, and phosphorus content. Soil N-acetyl- β- D-glucomannase (S-NAG) has a significant positive effect on alkaline phosphatase (λ= 0.641, *p* < 0.001), and all three enzymes had significant effects on microbial versatility (C, N, P: λ = - 0.827, 0.536, and 0.534, respectively). The results of the standard total effect (Fig. 5B) showed that AMF inoculation contributed the most to microbial multi-function, followed by fungal diversity. Soil β-glucosidase (S- β- GC) was negatively more correlated with microbial multi-function than soil N-acetyl- β- D-glucomannase (S-NAG).

## Discussion

### Microbial Community Diversity Promotes Soil Health and Plant Growth

The relative abundances of the 100 most abundant genera identified in previous studies served as the keystone microbial group of this study [28-30]. Due to the rapid development of microbial ecology, keystone microbial groups have been recognized as highly representative of the whole microbial community. Fungi are classified as Ascomycota (85 genera), Basidiomycota (10 genera), Mortierellomycota (1 genus), Rozellomycota (1 genus), and Chytridiomycota (3 genera). There is no doubt that Ascomycetes play a leading role in the phylogenetic relationship. The vigorous metabolic activities of these microorganisms can significantly improve the physical structure of the soil and make it more fertile.

The differences between inoculation of AMF alone and simultaneous inoculation of AMF and biochar were compared in this study by analyzing the soil's physical and chemical parameters and microbial community structure. Recently, biochar has attracted great interest due to its large surface area, heterogeneous pore structure, and various inherent minerals [31]. Biochar is rich in mineral elements (magnesium, potassium, nitrogen, and calcium) and can maintain soil pH, improving soil fertility and promoting plant growth [32]. It has been reported that biochar treatment can change root respiration and nitrogen and phosphorus uptake, thus improving plant growth, as shown in the overall improved growth performance [33, 34]. Similar to previous studies, AMF can influence the uptake and transport of soil elements by plants by increasing the contact area between roots and soil and regulating soil elements [35]. At the same time, we found that the addition of biochar can increase the colonization rate of AMF, revealing the influence mechanism of biochar and soil microorganisms based on the previous evidence that biochar can promote fungal activity. The results showed that AMF and biochar significantly increased the root length and surface area of plants (Table 1). Biochar and AMF inoculation induce complex and beneficial morphological, physiological and biochemical changes in plants, due to their involvement in soil solution modification [36-38]. It can be seen from the above results that the inoculation of AMF and biochar can increase the diversity of the rhizosphere microbial community and change the community structure, thereby providing a good environment for plant growth. To summarize, our results are consistent with a previous study that the combined application of biochar and AMF promoted plant growth [39].

### Effect of Keystone Microbial Diversity on Soil Nutrient Supply and Plant Absorption Capacity

Soil microorganism diversity is an essential indicator of soil health and plant fitness, as a wide range of microorganisms is involved in important soil functions. In this study, when the LDA threshold is set to 3.0, 35 fungal branches in each treatment group showed statistically significant differences (Fig. 2A). LEfSe analysis detected 16 biomarkers at the control group site, 16 at the treatment group site inoculated with AMF reagent, and only three at the treatment group site inoculated with AMF reagent and biochar (Fig. 2A). The branch diagram of the abundance from phylum to genus is shown in Fig. 2B; f_ Hypocreales_ fam_ *incertae_ sedis* dominated the control group. O_Hypocreales were predominant in the treatment group inoculated with AMF, and f_Pleosporaceae were predominant in the treatment group inoculated with AMF and biochar simultaneously.

The relative abundance of the microbial community in the treatment group inoculated with AMF and biochar was the most significant. The soil microbial community abundance is important for maintaining soil stability and versatility [40, 41]. Soil microorganisms increase the soil carbon content by influencing the carbon cycle, thus improving soil fertility and structure, allowing the soil to absorb and retain water and provide essential mineral elements and water for plant growth. Furthermore, the soil microbial community provides other nutrients plants need to improve their adaptability [42]. Soil can also maintain the balance of physiological functions in plants and promote healthy plant growth. According to the aforementioned findings, microbial community abundance is related to improving soil properties and material cycle and potentially benefits plant growth.

### Microbial Interaction Promotes Plant Root Development

Network analysis can be used to explore the symbiotic interaction of keystone microbial communities in complex environments, especially soil microbial communities [42, 43]. The microbial molecular ecological network shows different microbial community patterns. The keystone microbiota of the fungal community cooperates through a complex species interaction network regulated by the interaction of soil and plant factors.

Molecular ecological network analysis indicated that the combination of AMF and biochar treatment was responsible for the alteration in microbial communities. The control group network comprises 245 nodes and 6,681 edges (Figs. 3A-3C). The AMF treatment group network has 177 nodes and 3,031 edges, while the AMF and biochar treatment group network has 178 nodes and 5,306 edges. The control group network is the most complex and maintains a stronger structure. These nodes were regarded as key components in network, which help to maintain the stabilization of the molecular pathway [44].

The Zi-Pi diagram shows the ASV distribution of network nodes based on module topology (Figs. 3D-3F). The keystone species distributed in Connectors, Module hubs, and Network hubs are all marked in the corresponding network diagram. The control group (Fig. 3D) has two key nodes (unclassified_f_Sporocaceae, Cervicosporidium); the AMF treatment group (Fig. 3E) has five key nodes (Wallrothiella, Acrostalagus, Amphotometric, unclassified_f_Arteriae, Tetraclass); and the AMF and biochar treatment group (Fig. 3F) has two key nodes (Cutaneo trichoron, Microascus). All these nodes belong to connectors.

The module and network aggregation play an important role in maintaining the stability of microbial networks. The average clustering coefficient of the treatment group network inoculated with AMF and biochar is the lowest (Fig. 3G), and the stable network improves its resistance to external environmental interference. The modularity of the treatment group inoculated with AMF only was higher than that of the treatment group inoculated with both AMF and biochar, indicating that its fungal community was more resistant to environmental factors. Moreover, the fungus network of the AMF treatment group has a longer average path length, indicating that the interaction between network modules is stronger, allowing the fungal species to respond synchronously to environmental changes in the soil. Microbial diversity may be related to the complexity of symbiotic networks. The average connectivity of the AMF and biochar treatment group is the highest, indicating that the microbial community structure of the treatment group network is the most complex. Higher average connectivity increases network connectivity and resistance to environmental changes [45, 46].

Furthermore, the correlation matrix data revealed a significant correlation between AMF and the diversity of fungal communities, indicating that AMF played a key role in the network relationship of soil fungal communities. AMF regulates the structure and function of terrestrial ecosystems through co-growth with plant roots, enabling plants to colonize new habitats [11].

### Differences in the Functional Distribution of Soil Microbial Communities Affect the Material Cycle and Energy Transformation in Soil

The results showed significant differences in the functional distribution of soil microbial communities among different fungi. The histogram revealed that the AMF and biochar treatment group had the highest ASV abundance (Fig. 4B), followed by the control group (Fig. 4A), and the AMF treatment group had the lowest (Fig. 4C). There is a significant difference in 95% confidence interval between undefined saprotroph and animal pathogen–endophyte–lichen parasite–plant pathogen–wood saprotroph. The primary function of the soil microbial community is to promote material circulation, regulate soil ecological balance, and improve soil fertility, which are beneficial to plant growth. The different functional distributions of soil microbial communities affect the microecological balance of plant roots, resulting in different plant growth. Saprophytic fungi in the soil can decompose organic matter into carbon dioxide. *A. fistulosum* L. inoculation with AMF has increased nutrient content significantly. AMF inoculation increased crop uptake of essential nutrients (such as Ca, P, K, and other macronutrients).

### AMF Improves Microbial Diversity and Versatility, Thereby Affecting Plants

The structural equation model (SEM) was used to investigate how AMF affect the carbon, nitrogen, and phosphorus levels in soil, thus changing the diversity and versatility of the soil microbial community. The results showed that AMF significantly changed the relationship between soil carbon, nitrogen, phosphorus, microbial diversity, and versatility (Fig. 5A). Previous studies have shown that AMF can influence plant absorption and transportation of soil elements by secreting metabolites, increasing the root-to-soil contact area, and regulating the existing forms of specific soil elements [47]. The interaction between fungi significantly improves the versatility of microorganisms. Plant rhizosphere microorganisms and symbiotic microorganisms can directly provide plants with nitrogen, phosphorus and other mineral elements, promoting plant growth. SEM results confirmed that soil carbon, nitrogen, and phosphorus affect microbial versatility by regulating microbial diversity; however, the detailed potential mechanism of these impacts needs further study. The standard total effect results (Fig. 5B) showed that AMF inoculation contributed the most to microbial multi-function, followed by fungal diversity. Soil β-glucosidase (S- β- GC) is negatively correlated with microbial multi-function and is higher than soil N-acetyl- β- D-glucomannase (S-NAG). Table 1 shows that the AMF treatment group has the longest roots, while the AMF and biochar treatment group has the highest spore density. The carbon and phosphorus contents of the treatment group inoculated with AMF alone were lower than those of the treatment group inoculated with both reagents simultaneously.

Although there is no treatment group in this experiment that only adds biochar, the experiment is still persuasive. In our future research, we hope to investigate the specific impact of AMF-superimposed biochar on all aspects of plant growth more thoroughly. This study expands our knowledge of how AMF and biochar modify soil's carbon, nitrogen, and phosphorus content and fungal diversity, thus affecting the versatility of microorganisms.

## Conclusion

In conclusion, the diversity, structure, and functional characteristics of soil microbial communities in the AMF treatment differed significantly from those in the AMF + BC treatment. The microbial abundance was the highest in the AMF + BC treatment. AMF and biochar increased the differences in the functional distribution of soil microbial communities among different fungi. Finally, the SEM confirmed that AMF and biochar could improve the microbial multifunctionality by regulating the rhizosphere fungal community. AMF play a key role in the network relationship of soil fungal communities, influencing the carbon, nitrogen and phosphorus content, as well as the diversity of fungal community in the soil, which affects the versatility of microorganisms. These findings provide significant information about the response of rhizosphere fungi to AMF and biochar in soil, and offer a theoretical basis for the effects of AMF and biochar on plant growth and the mechanisms of rhizosphere microorganisms.

## Figures and Tables

**Table 1:** Plant growth of *Allium fistulosum* L. inoculated with or without AMF and biochar.

Treatments	Root plant height (cm)	Shoot plant height (cm)	Root fresh weight (g plant^-1^)	Shoot fresh weight (g plant^-1^)	Spore density (10 g^-1^)	AMF colonization intensity (%)	AMF colonization rates (%)	Total root length (cm)	Surface area (cm^2^)	Average diameter (mm)
CK	-	-	-	-	-	-	-	-	-	-
AMF	13.23 ± 0.55a	17.07 ± 1.21a	0.09 ± 0.04a	0.33 ± 0.03a	1.46 ± 0.05b	26.70 ± 0.29b	95.76 ± 1.64b	62.11 ± 27.01a	7.55 ± 2.99a	0.39 ± 0.02 b
AMF+BC	9.93 ± 1.18b	18.53 ± 5.41a	0.09 ± 0.01a	0.37 ± 0.19a	3.30 ± 0.03a	30.56 ± 0.62a	98.32 ± 0.68a	48.31 ± 10.14a	6.97 ± 0.82a	0.47 ± 0.05a

Treatments	Projected area (cm^2^)	Length/ volume (cm/m^3^)	Root volume (cm^3^)	Number of root tips	Bifurcation number	Crossing number	Soil β- Glucosidase (S- β- GC) (umol/d/g)	Soil N-acetyl- β- Dglucomannase (S-NAG) (umol/d/g)		Alkaline phosphatase (umol/d/g)
CK	-	-	-	-	-	-	27.98±0.34a	13.71±0.43a		9.79±0.10a
AMF	2.40 ± 0.95a	62.11 ± 27.01a	0.07 ± 0.03a	74 ± 44.84a	97 ± 86.69a	9.33 ± 8.08a	11.95 ± 2.04b	2.37 ± 0.37b		7.79 ± 0.05b
AMF+BC	2.22 ± 0.26a	48.31 ± 10.14a	0.08 ± 0.003a	66.33 ± 31.50a	97 ± 71.13a	7 ± 6.08a	9.80 ± 1.28b	3.03 ± 0.18b		3.62 ± 0.07c

Data (mean ± SD, *n* = 4) followed by different letters in the same column indicate significant (*p* < 0.05) differences.
